# The *Aspergillus nidulans* Peripheral ER: Disorganization by ER Stress and Persistence during Mitosis

**DOI:** 10.1371/journal.pone.0067154

**Published:** 2013-06-24

**Authors:** Ane Markina-Iñarrairaegui, Areti Pantazopoulou, Eduardo A. Espeso, Miguel A. Peñalva

**Affiliations:** Departamento de Biología Celular y Molecular, Centro de Investigaciones Biológicas Consejo Superior de Investigaciones Científicas, Madrid, Spain; University of Wisconsin - Madison, United States of America

## Abstract

The genetically amenable fungus *Aspergillus nidulans* is well suited for cell biology studies involving the secretory pathway and its relationship with hyphal tip growth by apical extension. We exploited live-cell epifluorescence microscopy of the ER labeled with the translocon component Sec63, endogenously tagged with GFP, to study the organization of ‘secretory’ ER domains. The Sec63 *A. nidulans* ER network includes brightly fluorescent peripheral strands and more faintly labeled nuclear envelopes. In hyphae, the most abundant peripheral ER structures correspond to plasma membrane-associated strands that are polarized, but do not invade the hyphal tip dome, at least in part because the subapical collar of endocytic actin patches constrict the cortical strands in this region. Thus the subapical endocytic ring might provide an attachment for ER strands, thereby ensuring that the growing tip remains ‘loaded’ with secretory ER. Acute disruption of secretory ER function by reductive stress-mediated induction of the unfolded protein response results in the reversible aggregation of ER strands, cessation of exocytosis and swelling of the hyphal tips. The secretory ER is insensitive to brefeldin A treatment and does not undergo changes during mitosis, in agreement with the reports that apical extension continues at normal rates during this period.

## Introduction

A remarkable aspect of the biology of filamentous fungi is their ability to grow exclusively by apical extension. *Aspergillus nidulans* is a useful experimental system to study cell biological processes due to its amenability to classical and reverse genetic analyses and to its suitability to live microscopy studies [1]. A fundamental question is how growth of hyphal tip cells is maintained sustainably. Such polarity maintenance [2] is critically dependent on exocytosis. Exocytic carriers fueling tip growth are mainly, if not exclusively, targeted to the apex [3], [4], [5]. Indeed hyphal tip cells contain, underneath the apical plasma membrane, an idiosyncratic structure denoted the Spitzenkörper, where secretory carriers accumulate before their fusion with the plasma membrane [6], [7], [8].

The spatial organization of the classical secretory pathway reflects the needs imposed by the polarization of exocytosis. Both the late and the early Golgi cisternae show strong polarization, predominating ahead of the leading nucleus [9], [10], [11]. In agreement with the accepted view that early Golgi cisternae form by coalescence of ER-derived COPII carriers [12], ER exit sites are also polarized, as it is for the ER in another *Aspergillus* species [13], although to a lesser extent than the Golgi [9]. Thus, assuming that the distribution of the ER ultimately contributes to the overall polarization of the secretory pathway, the key issue is what determines the ER polarization. In *Saccharomyces cerevisiae*, the ER includes two different ‘domains’ [14], namely nuclear envelopes (NEs) and ‘peripheral’ ER, the latter including all ER strands excepting the NEs. The largest ‘subdomain’ of the peripheral ER is composed of plasma membrane (pm)-associated cortical ER sheets [15]. Hyphal tip growth is maintained during mitosis [16], indicating that the exocytic compartments remain functionally active during this period. Indeed the Golgi is not disorganized during mitosis [9], indicating that ER function is sufficiently preserved. As the *A. nidulans* nuclear envelopes (NEs) remain intact during mitosis [17], [18], mitotic ER function is attributable, at least in part, to the NE domains; however, the potential changes undergone by peripheral ER domains have not yet been analyzed. In their seminal studies of the ER in filamentous fungi, carried out with the basidiomycete *Ustilago maydis*, Wedlich-Soldner *et al.* used an ER-targeted GFP construct carrying a HDEL ER retention signal. This luminal fluorescent protein probe labeled the ER as well as vesicles mediating early-Golgi-to-ER recycling [19], and thus is not ideally suited to study secretory ER domains.

Therefore, to study the *A. nidulans* ER secretory domains we chose a component of the ER translocon. The term ‘translocon’, coined by Walter and Lingappa [20], denotes the ‘pores’ and the molecular engines mediating the delivery of eukaryotic secretory proteins synthesized in the cytosol into the ER lumen. Studies in *S. cerevisiae* have been instrumental to understand the mechanistic and genetic basis of translocation. These studies showed that the composition of the translocon varies depending on whether translocation occurs co-translationally or post-translationally. A core component of all translocons is the Sec61 heterotrimer, composed of Sss1, Sbh1 and the pore-forming subunit Sec61 [21]. Post-translational translocation, the pathway that is seemingly followed by a larger fraction of proteins in fungi [21], is mediated by the so denoted ‘SEC complex’ containing, in addition to the Sec61 heterotrimer, the Sec62/63 complex including Sec62, Sec63, Sec71 and Sec72 [22], [23]. Co-translational translocation requires, *in vitro*, the Sec61 complex and the signal recognition particle (SRP) receptor [24]. However Sec63 appears to be involved in all translocon complexes *in vivo*, a conclusion strongly supported by the finding that a SEC’ complex containing all SEC components excepting Sec62 is required for co-translational translocation [25]. In view of this and of the fact that Sec63 is an integral membrane protein localizing to membrane domains, not to the ER lumen, we set out to study secretory ER domains using Sec63 as marker.

Here we show that the ER structure is dramatically altered by reductive stress, correlating with cessation of exocytosis. We show that the secretory ER, like NEs, remains intact through mitosis. Lastly we show that F-actin plays a key role in maintaining the polarization of the ER, which is characteristically associated with the subapical ring of endocytic patches.

## Materials and Methods

### *A. nidulans* Media and Molecular Genetics

*A. nidulans* was cultured on complete medium (MCA) and synthetic complete medium (SC) containing 1% glucose and 5 mM ammonium tartrate (*i.e.* 10 mM NH_4_^+^) as carbon and nitrogen source, respectively [26]. Endogenous GFP tagging of Sec63 (AN0834) was made with a cassette containing *A. fumigatus pyrG* (*pyrG^Af^*), constructed by fusion PCR [27]. GFP was attached to the C-terminal residue of Sec63 by means of a (Gly-Ala)5 linker. The recipient strain for transformation [28] carried *pyrG89* (resulting in pyrimidine auxotrophy) to allow selection of primary transformants and a *nkuA*Δ mutation to prevent non-homologous recombination [29]. The correct recombination event was confirmed by Southern blotting. The complete genotypes of the strains used in this work are as follows: MAD1399 [30], *pyrG89 yA2 abpA::mRFP::pyrG^Af^ pabaA1*; MAD2173, *pyrG89 argB2 pyroA4* Δ*nku::argB sec63::gfp::pyrG^Af^*, MAD2077 [31], *pyrG89 pyroA4* Δ*nku::bar synA::GFP::pyrG^Af^*; MAD2518, *pyrG89 yA2 abpA::mRFP::pyrG^Af^; sec63::gfp::pyrG^Af^*; MAD2519, *pyrG89 wA4 inoB2 pyroA4* Δ*nku::bar hhoA::mCherry::pyroA^Af^ sec63::gfp::pyrG^Af^ pacC900*; MAD2982, pyrG89 argB2? pyroA4::[pyroA*-gpdA^mini^::mRFP-PH^OSBP^] nkuAΔ*::argB sec63::gfp::pyrG^Af.^*

#### Microscopy

Germlings and hyphae were cultured at 28°C in Lab-Tek chambers (Thermo Fischer Scientific; 0.3 ml of medium per well) at 25–28°C in pH 6.5 'watch minimal medium' (WMM) [32] containing 0.1% glucose and 5 mM ammonium tartrate as sole carbon and nitrogen source, respectively. When indicated, dithiothreitol (DTT) was added at 8 mM to induce UPR. Latrunculin B was added at 100 µM to depolymerize F-actin [9]. Brefeldin A was used at 300 µg/ml [9]. Benomyl was used at 4.8 µg/ml, a concentration that led to depolymerization of MTs within 10 min, as determined with a control strain expressing GFP-α-tubulin (TubA) [16].

Images were acquired with a Leica DMI6000B inverted fluorescence microscope driven by Metamorph® software (Molecular Dynamics), coupled to an ORCA ER-II (Hamamatsu) camera. Excitation of fluorescent proteins was achieved with an external light source equipped with a metal halide lamp for epifluorescence excitation (Leica, EL6000). The microscope was also equipped with GFP (Semrock GFP-3035B) and mRFP/mCherry (Semrock TXRED-4040) filter sets, and a HCX 63×1.4 NA objective (Leica 11506187). To minimize photodamage, the excitation light attenuator was set to the minimal position giving a reasonable intensity of fluorescence. Maximal intensity projections were obtained from z-stacks of images that had been contrasted with the ‘unsharp mask’ filter of Metamorph (settings: filter width, 9 pixels; scaling factor 0.75; result scale 2) or deconvolved using Huygens Software (Hilversum, The Netherlands). Time-lapse sequences (5 dimensions: x, y, z, t, red and green channels) were obtained from maximal intensity projections of deconvolved single time point z-stacks. Images were converted to 8-bit greyscale (and usually shown in inverted contrast) or to 24-bit RGB, and annotated with Corel Draw (Corel). Time-lapse sequences were converted to QuickTime using ImageJ 1.37 (http://rsb.info.nih.gov/ij/).

## Results and Discussion

### Morphology of the*A. nidulans* ER Visualized with Endogenously Tagged Sec63

**Figure 1:**
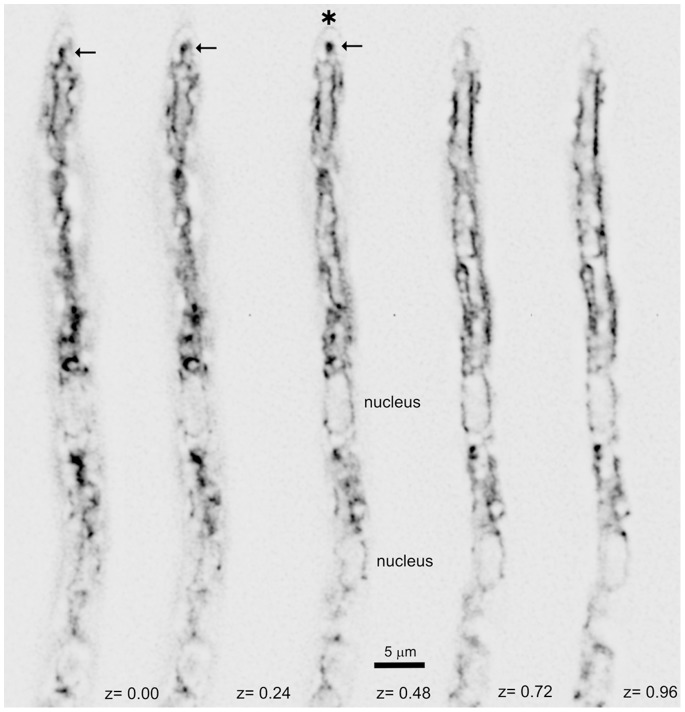
The ER visualized with Sec63-GFP. Different planes (z coordinates indicated in µm) of z-stack of images of a hyphal tip cell. The position of the nucleus is indicated in the middle plane. The asterisk indicates the apical border of the hypha, whereas the arrow indicates the apex-proximal ER protusion.

To visualize the *A. nidulans* ER we GFP-tagged endogenously Sec63, a key component of the protein complex mediating translocation of newly synthesized proteins into the ER [33]. Sec63 corresponds to AN0834 (www. aspgd.org). Strains carrying *sec63-gfp* displayed wild-type growth, showing that the fusion protein is functional. z-stacks of Sec63-GFP hyphal tip cell images, processed with a deconvolution algorithm to remove out-of-focus signal, provided a very detailed view of the ER. Sec63-GFP localizes to a network of ‘strands’ corresponding to the different ER ‘domains’ reported in yeast [14], namely nuclear envelopes (NEs) and ‘peripheral’ ER, including pm-associated ER strands and a faint network of interconnecting tubules (Figure 1). NEs were only faintly labeled with Sec63, which contrasts with the strong labeling of NEs by Erg24 reported previously [34]. Like in *S. cerevisiae* [15], cortical strands associated to the plasma membrane represented the largest peripheral ER subdomain (Figure 1). These cortical strands were polarized, as described for *A. oryzae* [13] and for dikaryotic hyphae of *U. maydis* [19]. Peripheral ER strands were excluded from the apical dome (Figure 1). In the tip region the ER network was frequently capped by a finger-like protrusion (arrowed in Figure 1) directed towards, but not reaching the apex (apex indicated by an asterisk).

**Figure 2:**
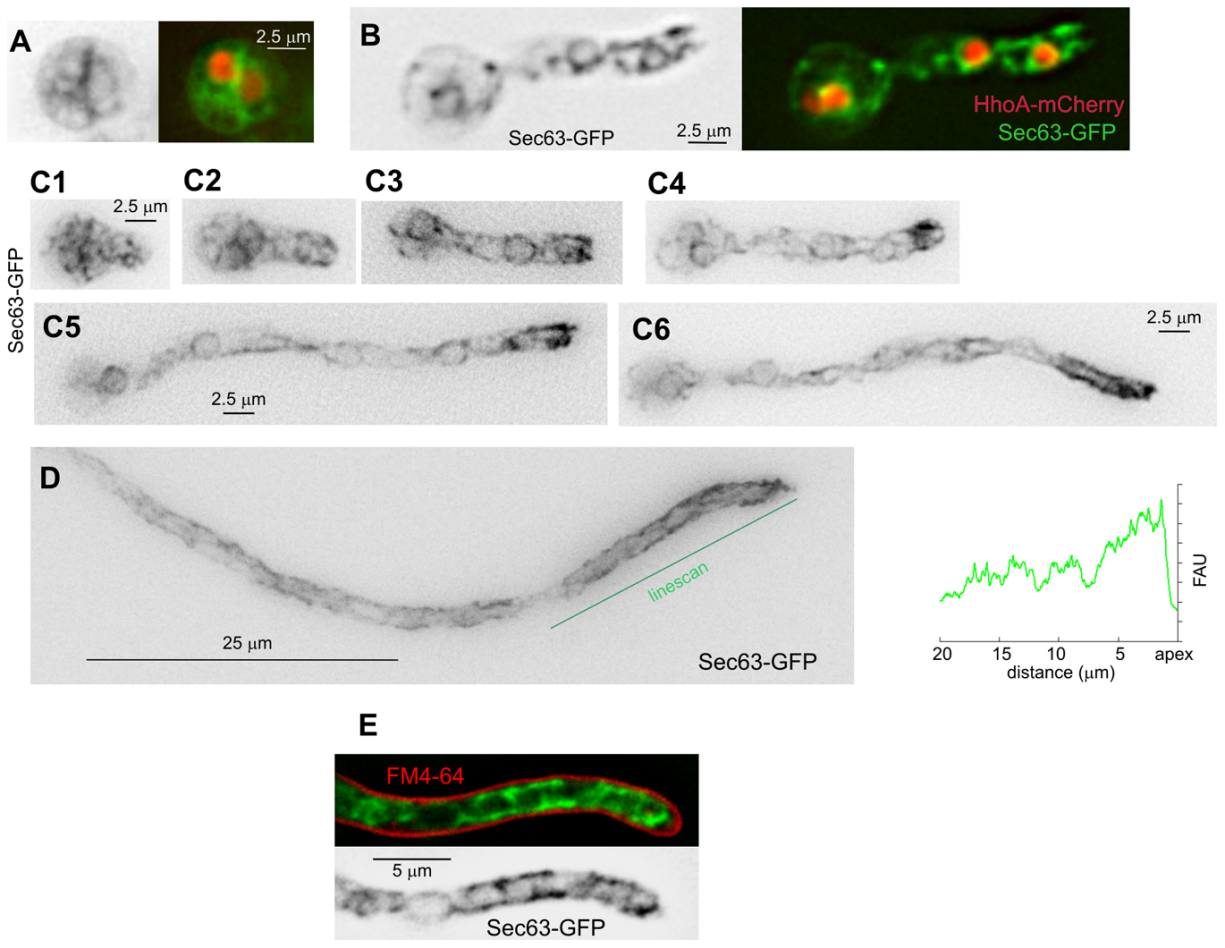
Changes in the ER in swollen conidia, germlings and mature hyphae. **A.** Swollen conidium after the first mitotic division. Sec63-labelled ER is shown on the left in inverted grey contrast. The right image is a merge of the Sec63-GFP (green) and HhoA-mCherry (that labels nuclei/chromatin) channels**.** **B.** A germling, with fluorescent markers displayed as in **A. C.** Sec63-GFP ER (inverted contrast) in germlings imaged at different stages after polarity establishment (all images displayed at the same magnification). Peripheral ER strands concentrated near the tip are visible at the stages shown in **C3** through **C6**. The prominence of the tip pm-associated ER strands increases with the length of the germtube. **D.** Long hypha. A linescan of the Sec63-GFP signal across the indicated line is shown on the right (FAU, fluorescence arbitrary units). **E.** Cortical ER strands do not overlap with the plasma membrane, stained with FM4-64.

The relative importance of the different domains varied depending on whether short germlings or long hyphae were examined (Figure 2). In conidiospores growing isotropically, and in germlings photographed shortly after establishing polarity, the ER appeared as a faint reticulate network in which NEs were hardly noticeable without the aid of a nuclear marker (Figure 2, A and B). NEs became more visible after nuclei moved into the germtube (Figure 2B). At approximately this stage, strands associated to the plasma membrane became prominent in the proximity of the tip; more so as apical extension proceeded (Figure 2C, C1 through C6), until reaching the stage of long hyphae. In tip cells of long hyphae, polarized pm-associated strands became the most abundant Sec63-containing ER structures (Figures 1 and 2D). These strands do not overlap with the plasma membrane labeled with FM4-64 (Figure 2E). Given that long hyphae grow ∼5 times faster than germlings [16], and that cortical strands generally correspond to ER sheets enriched for poly-ribosomes and translocation complexes [14], the prominence and polarization of pm-associated ER strands in hyphal tip cells possibly reflects the dependence that these have on secretion for rapid apical extension. Video S1 shows the dynamics of the ER network over a 30 min period (time resolution, 2 frames/min), illustrating the prominence of the polarized pm-associated strands as growth by apical extension proceeds. Video S2 shows a high time-resolution series (2.5 frames/sec over a 30 sec period) displaying the short-term dynamics of the ER strands.

### The Subapical Actin Collar Prevents the ER from Invading the Apical Dome

**Figure 3:**
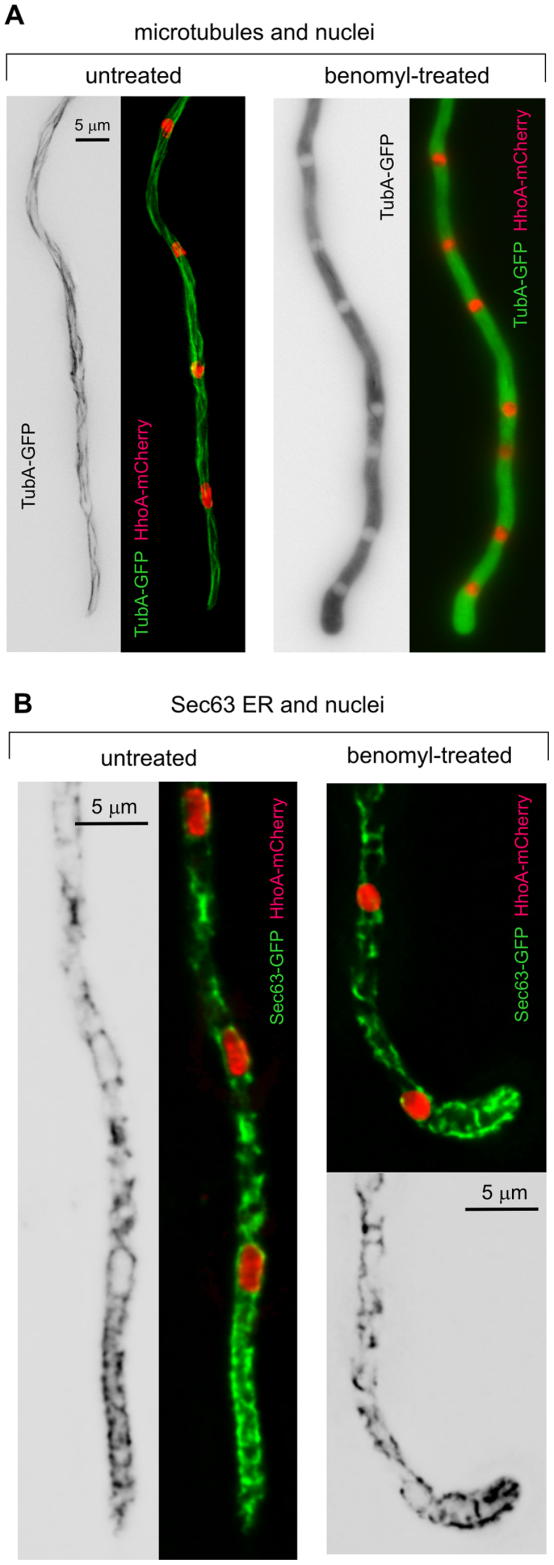
The ER does not undergo major changes following microtubule depolymerization. **A.** Control experiment demonstrating the depolymerization of MTs (green) upon treatment with benomyl. Unpolymerized TubA-GFP accumulates in the cytosol, leaving the nuclei empty (nuclei are labeled with HhoA-mCherry). The picture was taken after after 17 min of incubation in the presence of the drug. **B.** Effect of benomyl treatment on the Sec63-GFP-labeled ER. The representative example shown on the right corresponds to a cell incubated for 30 min in the presence of benomyl. The positions of the nuclei are revealed by the HhoA-mCherry fluorescence.

The organization of the ER in the tip region was consistent with a physical barrier, perhaps elements of the cytoskeleton, impeding strands to move forward. We tested this idea with anti-microtubule (MT) and anti-actin treatments. Benomyl (used at 4.8 µg/ml) led to the rapid (within 10 min) depolymerization of MTs, as determined with a control strain expressing TubA-GFP (Figure 3A). Under equivalent conditions, benomyl affected the structure of the ER network, which was less organized. However, ER strands maintained their polarization, accumulating in the tip region, ahead of the leading nucleus (Figure 3B; note the characteristic loss of directionality resulting from MT depolymerization in the benomyl-treated hyphal tip cell [16]).

**Figure 4:**
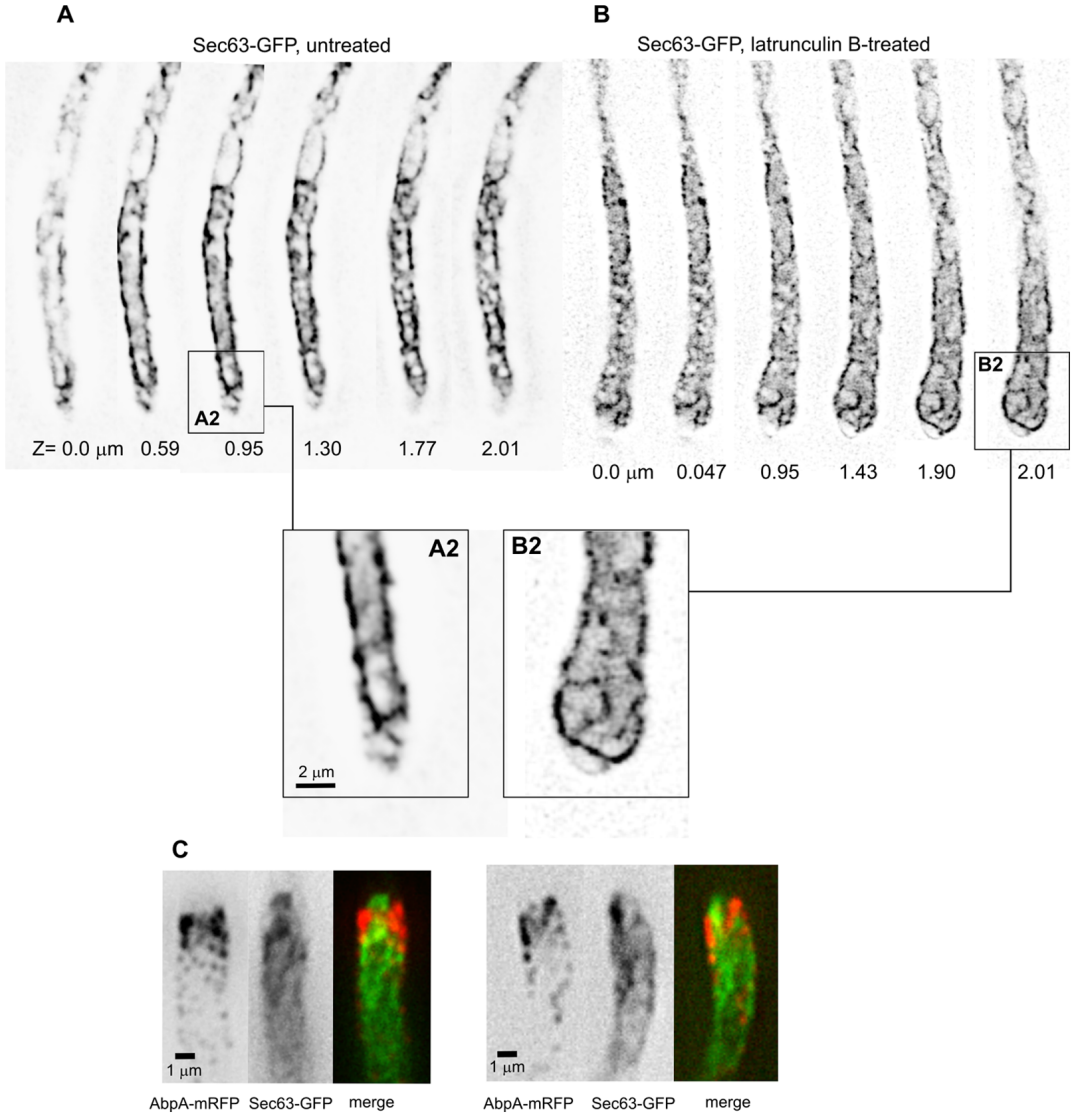
Role of F-actin and of the subapical collar of actin patches in determining peripheral ER morphology. **A.** Individual images of a z-stack of Sec63-GFP in an untreated hyphal tip cell control. **B.** Individual images of a z-stack of Sec63-GFP in a hyphal tip cell treated with latrunculin B. **A2** and **B2** correspond to the rectangular regions indicated in **A** and **B**, respectively, shown at double magnification. **C.** Dual-channel images of Sec63-GFP and mRFP-AbpA. Two examples of hyphal tip cells are shown. Images represent maximal intensity projections of z-stacks treated with an ‘unsharp’ filter.

In contrast, the effect of anti-actin treatment was striking. Upon incubation with latrunculin B, the ER underwent major reorganization, adopting a more regular reticulate structure connecting internal and cortical strands (Figure 4, panels A and B). This result suggested strongly that F-actin scaffolds the ER structure. More importantly, the ER strands invaded the swollen latrunculin B-treated tips, and the apical ER protrusion was no longer seen, suggesting that the organization of the ER in the tip region involves a ‘barrier’ dependent on F-actin (Figure 4, B2). An obvious candidate to form this barrier was the subapical endocytic collar of actin patches [3], [30], [35]. Thus, we co-imaged Sec63-GFP with the actin patch marker AbpA-mRFP. These images showed that the position of the endocytic collar is consistent with this interpretation (Figure 4C). Moreover, the ER protrusion and the endocytic collar showed complementary shapes in maximal intensity projections, strongly indicating that the endocytic collar actually constricts the ER (Figure 4C). Therefore, the cluster of F-actin-containing patches forming the endocytic collar, or structures associated to it, likely act(s) as a physical barrier excluding the ER from the apical dome.

### Secretion Arrest by DTT-induced UPR

**Figure 5:**
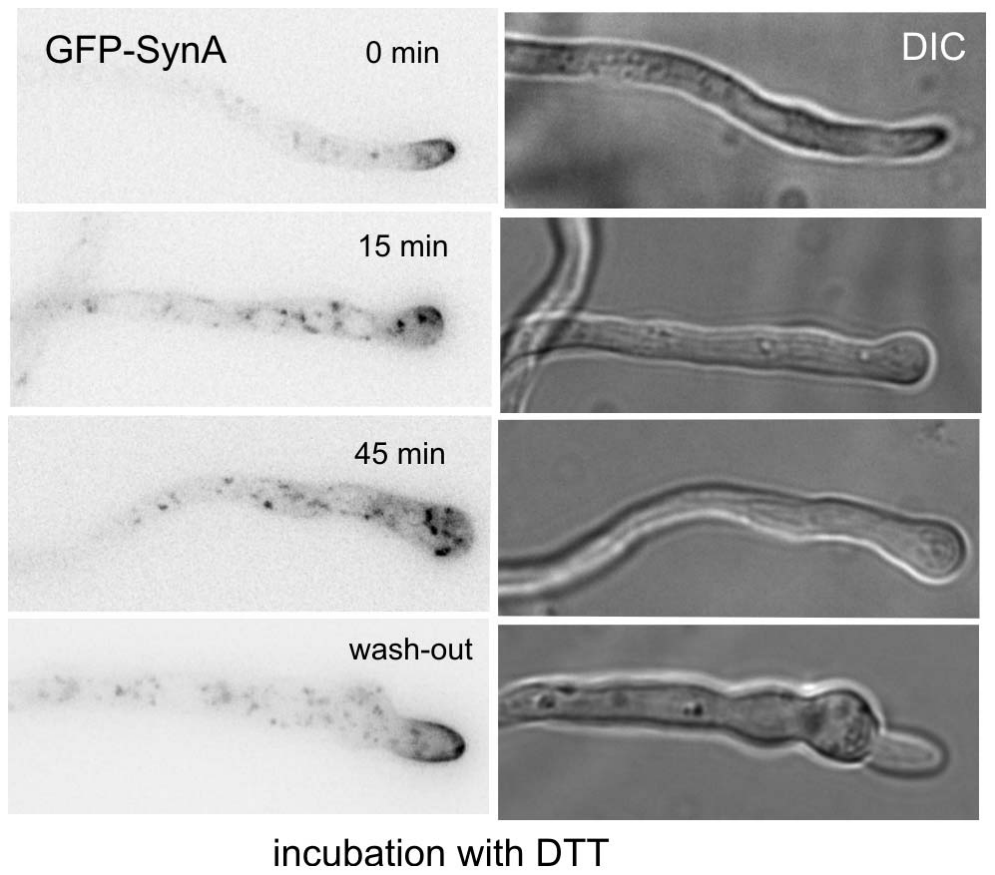
Reductive ER stress results in reversible impairment of exocytosis. GFP-SynA (inverted greyscale) and Nomarski (DIC) images of hyphal tips of cells incubated with DTT for the indicated times. The wash-out tip example corresponds to a DTT-treated cell further incubated in medium lacking the reducing agent.

DTT has been shown to result in ER stress and unfolded protein response (UPR) in yeast and filamentous fungi, including *A. nidulans* [36], [37], [38], [39]. We asked whether we could detect ER stress morphologically at the subcellular level, exploiting that activation of the UPR should block ER exit, thus leading to an arrest in secretion. The v-SNARE SynA^Snc1^ is a useful marker for secretion because SynA^Snc1^ traveling with secretory carriers critically contributes to the tetrahelical SNARE bundle mediating docking and fusion of these carriers with the plasma membrane. Like its *Saccharomyces cerevisiae* orthologues Snc1p, SynA^Snc1^ is strongly polarized by endocytic recycling, localizing to the apical dome and to the Spitzenkörper [3], [10], [31], [40]. Impairment of the secretory function of the ER would be expected to result in tip swelling and mislocalization of SynA^Snc1^, resembling the effects of the Golgi-disorganizing drug brefeldin A [9]. Figure 5 demonstrates that this is indeed the case. DTT treatment resulted in tip swelling and detectable SynA^Snc1^ mislocalization within 15 min, both effects being even more marked after 45 min. Tip growth was restored, as were the normal morphology of the tip and the normal localization of SynA^Snc1^ (to an apical crescent and the Spitzenkörper), after washing out DTT (Figure 5). Collectively these data show that induction of UPR results in a sharp yet reversible arrest in secretion.

### UPR Induces ER Aggregation

**Figure 6:**
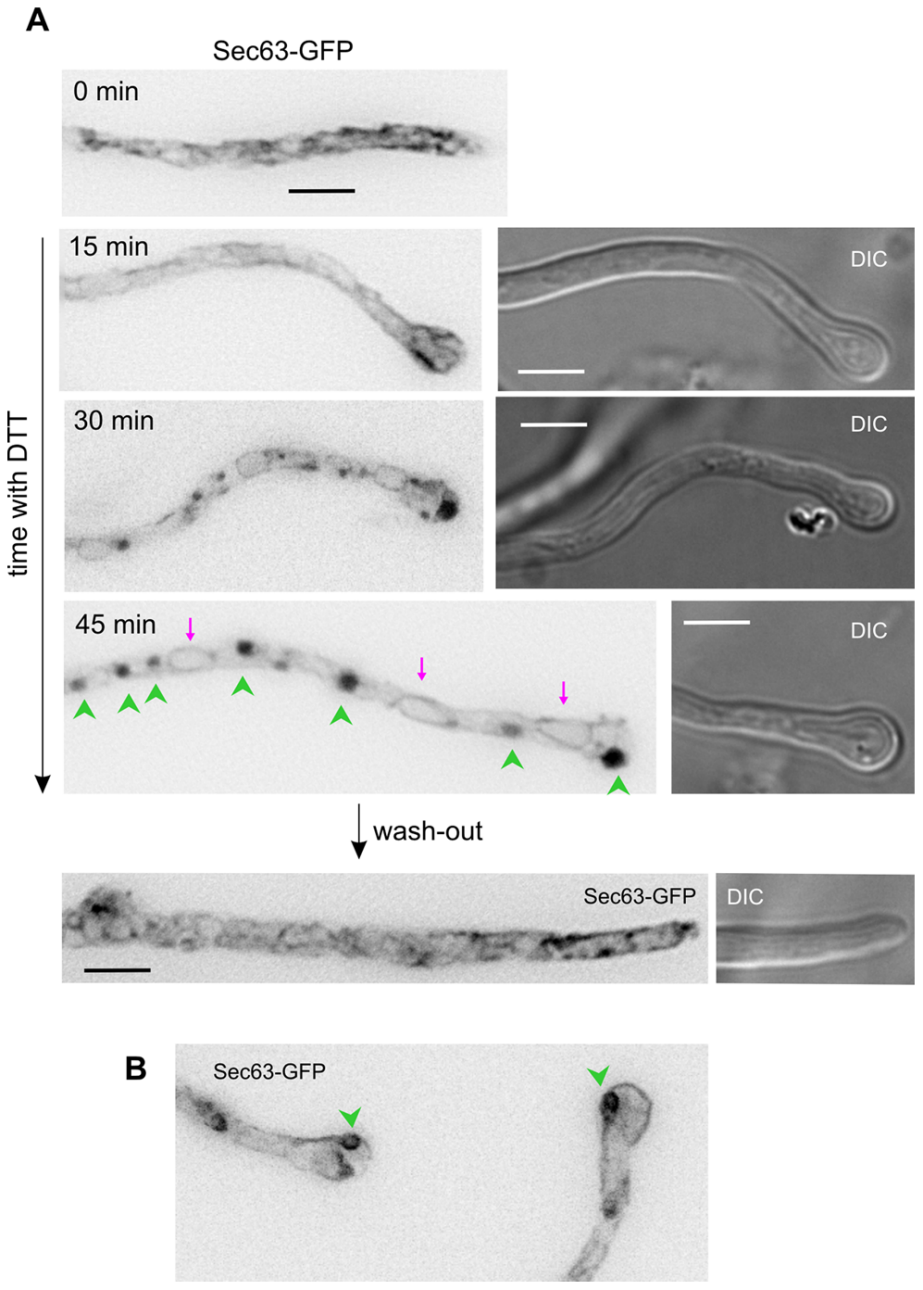
Reductive stress leads to aggregation of peripheral ER strands. **A.** Sec63-GFP images (inverted greyscale) of cells treated with DTT for the indicated time periods. Images correspond to maximal intensity projections of z-stacks processed with an ‘unsharp’ filter. In the 45 min GFP image, green arrowheads indicate aggregates of ER strands and magenta arrows indicate nuclei, whose NEs, unlike peripheral ER strands, appear unaltered. All images are shown at the same magnification. Bars, 5 µm. **B.** Examples of ring-shaped ER aggregates in cells treated with DTT for 130 min.

Next, we examined the effects of DTT treatment on the ER structure. Marked disorganization of the pm-associated ER at the tip was noticeable after 15 min of treatment, a time at which tip swelling was already conspicuous (Figure 6A). Following a 30-min incubation with DTT, abnormal aggregates of ER membranes became noticeable in the hyphal tips (Fig. 6A). After 45 min, aggregates also formed in regions distant to the tip (Fig. 6A, arrowheads), indicating that the extent of ER aggregation depends on the time of incubation in reductive stress conditions. In some examples, examination of individual planes of Z-stacks revealed that ER strands in these aggregates formed donought-like structures (Figure 6B). All these changes were reversible upon washing out the reducing agent, demonstrating that this treatment does not result in cell death. Of note, the NE sheets appeared unaffected by DTT/UPR (red arrows; Figure 6A). Thus, DTT-induced UPR results in a subcellular response leading to the collapsing and aggregation of the cortical ER but not of NEs. This is consistent with the view that cortical ER strands in which Sec63 (*i.e.*, the translocon complexes) predominates correspond to the most active ER subdomain in terms of secretion.

**Figure 7:**
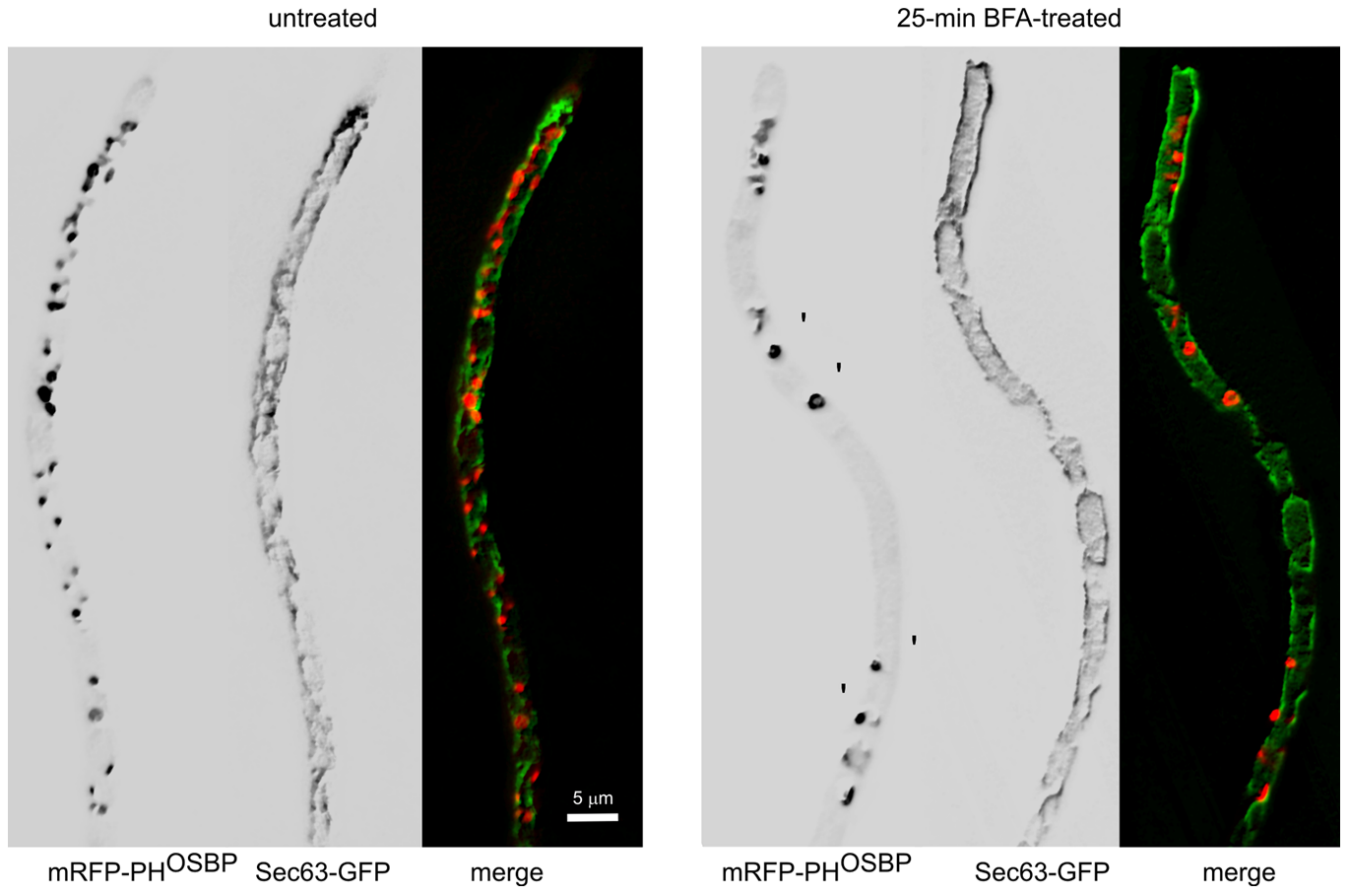
The ER is brefeldin A-insensitive. A strain coexpressing Sec63-GFP with the late Golgi marker mRFP-PH^OSBP^ was treated with brefeldin A for 25 min. Under these conditions, late Golgi cisternae form aggregates. In contrast, the ER appears to be largely resistant to the drug. Images represent maximal intensity projections of z-stacks of deconvolved images.

In contrast to the marked effects of DTT, we demonstrated that the Golgi-targeting drug brefeldin A (BFA) does not affect the ER. BFA treatment leads to aggregation of Golgi cisternae into larger ‘brefeldin bodies’ (Fig. 7; note the aggregation of late Golgi cisternae labeled with mRFP-PH^OSBP^) [9], [10], [41]. However, the ER, both NE and peripheral, was unaffected (Fig. 7), even though the hyphal tips swelled due to secretion impairment. Thus, the *A. nidulans* ER is BFA-resistant and the effects of BFA are restricted to the Golgi.

### The Peripheral ER does not Undergo Changes during Mitosis

**Figure 8:**
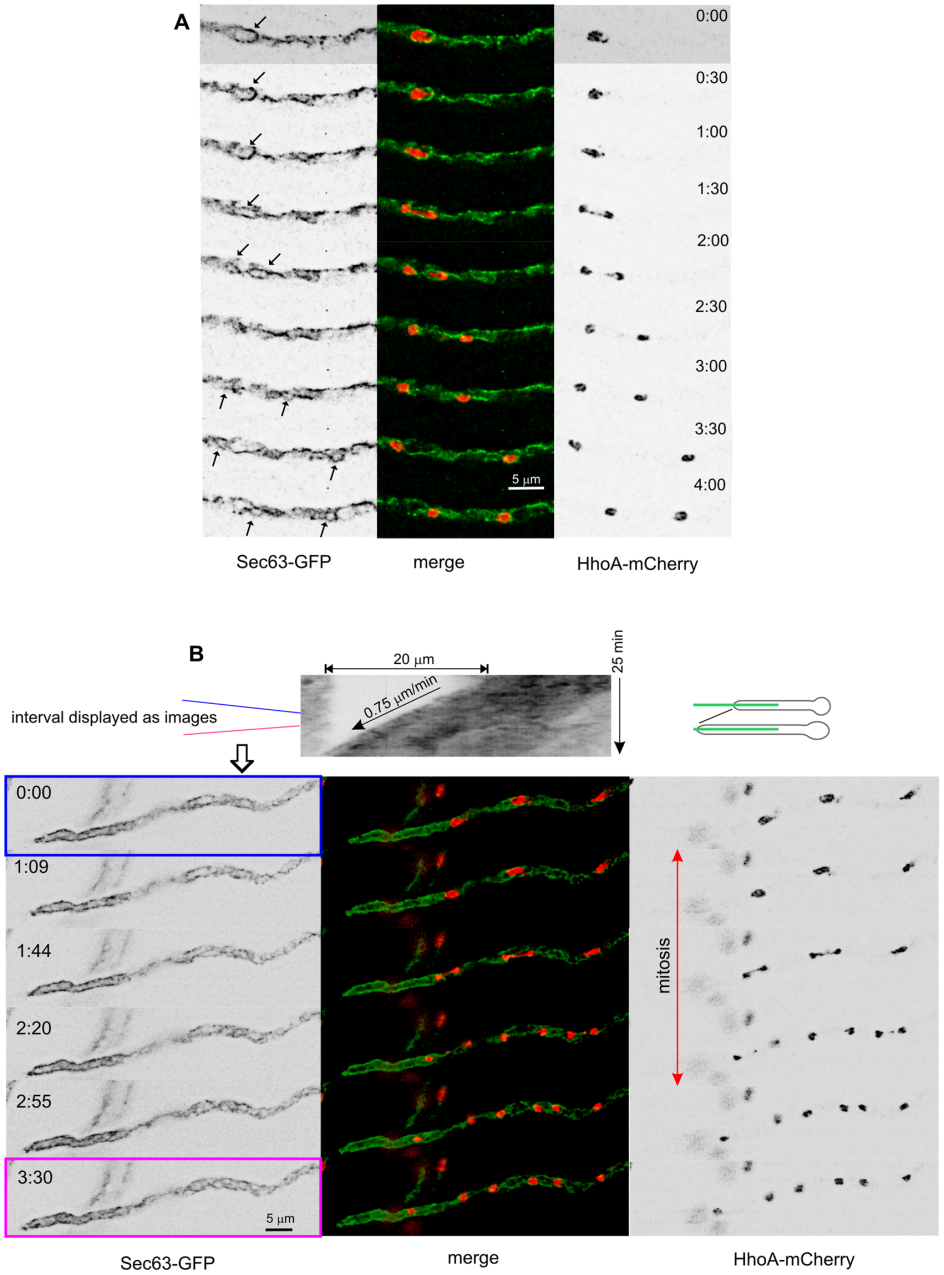
The peripheral Sec63 ER is not disorganized during mitosis. **A.** Nuclear division in a tip-distant region of a cell coexpressing Sec63-GFP and HhoA-mCherry (‘green’ and ‘red’ channels displayed in inverted greyscale). Time is indicated in min:sec. Whenever possible, the position of the intact NEs of the dividing nuclei is indicated with arrows. **B.** The peripheral ER does not undergo disorganization during mitosis. The top greyscale image is a kymograph displaying growth of the hyphal tip cell shown below. The kymograph was traced as schematized on the right. The series of images displayed below the kymograph (Video S3 should be consulted) show that the peripheral ER does not undergo rearrangements during mitosis.

The fate of NE sheets during *A. nidulans* mitosis has been extensively studied by Ukil *et al.*, who used fluorescent Erg24 (a ‘general’ ER marker) to show that a double NE constriction generates, in addition to the two daughter nuclei, a nucleolar remnant that is later disassembled [34]. To examine the fate of peripheral ER subdomains during mitosis, we collected time-lapse series of cells coexpressing Sec63-GFP with HhoA^HH1^-mCherry, to label chromatin. As noted above, due to their relatively faint staining with Sec63 the NEs of nuclei undergoing mitosis were hardly traceable against the background of peripheral strands (Figure 8A; NEs arrowed when possible). In contrast, pm-associated strands enriched in translocon (*i.e.*, Sec63) complexes were very conspicuous all throughout mitosis. Figure 8B (Video S3) shows a hyphal tip cell undergoing mitosis, growing at 0.78 µm/min. The kymograph shows that the rate of apical extension does not change even though the three nuclei shown undergo mitosis. The sequence clearly shows that the peripheral ER and, in particular, the polarized pm-associated strands, are not affected to any detectable extent by mitosis (Figure 8B).

Polarity maintenance requires that the growing regions of hyphal tip cells be populated by secretory organelles. Our data showing that polarized pm-associated ER strands are intimately connected with the F-actin-containing subapical endocytic collars would be consistent with the ‘tip’ of the ER strands being carried along with the hyphal tip as apical extension occurs. Whether this occurs simply by ‘constriction’ of the ER by the collar of actin patches and/or by proteins associated to it, or whether there is involvement of proteinaceous apical polarity landmarks [42], of septin-dependent diffusion barriers/structural elements [43], [44], [45], or even of lipid domains associated with the tip establishing direct contact with the ER strands, are important questions for the future.

In mammalian cells the peripheral ER, unlike NEs, does not disassemble during mitosis but undergoes major changes in structure correlating with detectable inhibition of ER export [46], [47]. Yeasts of the basidiomycete *U. maydis* display a particular way of ‘open mitosis’ in which the nuclear envelope is broken-down [48], which leads to distortion of the ER network [19]. The biology of *A. nidulans*, like that of other filamentous ascomycetes, is conditioned by the compulsory need of its hyphae to grow by apical extension. Apical extension is crucially dependent on exocytosis, which implies that, during vegetative growth, the secretory pathway cannot be compromised under any circumstances. Apical extension is not slowed down by mitosis [16], [49]. Thus, the demonstration that impairment of ER function results in cessation of growth and tip swelling (this work) leads to the prediction that the peripheral ER should not undergo major reorganizations during this period. We have thoroughly documented that this is the case. Thus, mitosis does not result in NEs disassembly [17], [34]; it does not affect the peripheral ER either (this work) and, consequently, is does not affect the Golgi [9]. In summary, the full capacity of the *A. nidulans* classical secretory pathway is maintained during mitosis. This, together with the persistence during mitosis of a tip-associated subpopulation of cytoplasmic MTs that possibly contribute to the targeting of secretory carriers to the tip, provide the fungus with the basic tools to maintain apical extension under mitotic circumstances [16], [49].

## Supporting Information

Video S1 **Dynamics of the Sec63-labeled ER in growing hyphae.** Sequences were assembled from deconvolved z-stacks of Sec63-GFP images covering growth of several hyphal tip cells over a 30 min period. Time is in min:sec.
(MOV)

Video S2 **Short-term dynamics of the Sec63-labeled ER.** Single-plane stream acquisition (2.5 frames/sec) of Sec63-GFP images.
(MOV)

Video S3 **Peripheral ER strands do not undergo rearrangements during mitosis.** Top, Sec63-GFP (inverted greyscale); bottom, HhoA-mCherry (inverted greyscale); middle, merge of the two channels. Time is in min:sec.
(MOV)

## References

[1] Peñalva MA, Galindo A, Abenza JF, Pinar M, Calcagno-Pizarelli AM, et al (2012) Searching for gold beyond mitosis: mining intracellular membrane traffic in *Aspergillus nidulans* . Cell Logist 2: 2–14. 22645705 10.4161/cl.19304 PMC3355971

[2] Momany M (2002) Polarity in filamentous fungi: establishment, maintenance and new axes. Curr Opin Microbiol 5: 580–585. 12457701 10.1016/s1369-5274(02)00368-5

[3] Taheri-Talesh N, Horio T, Araujo-Bazán LD, X., Espeso EA, Peñalva MA, et al (2008) The tip growth apparatus of *Aspergillus nidulans* . Mol Biol Cell 19: 1439–1449. 18216285 10.1091/mbc.E07-05-0464 PMC2291424

[4] Taheri-Talesh N, Xiong Y, Oakley BR (2012) The Functions of Myosin II and Myosin V Homologs in Tip Growth and Septation in *Aspergillus nidulans* . PLoS ONE 7: e31218. 22359575 10.1371/journal.pone.0031218 PMC3281053

[5] Kohli M, Galati V, Boudier K, Roberson RW, Philippsen P (2008) Growth-speed-correlated localization of exocyst and polarisome components in growth zones of *Ashbya gossypii* hyphal tips. J Cell Sci 121: 3878–3889. 18984630 10.1242/jcs.033852

[6] Harris SD, Read ND, Roberson RW, Shaw B, Seiler S, et al (2005) Polarisome meets Spitzenkörper: microscopy, genetics, and genomics converge. Euk Cell 4: 225–229. 10.1128/EC.4.2.225-229.2005 PMC549335 15701784

[7] Hohmann-Marriott MF, Uchida M, van de Meene AM, Garret M, Hjelm BE, et al (2006) Application of electron tomography to fungal ultrastructure studies. New Phytol 172: 208–220. 16995909 10.1111/j.1469-8137.2006.01868.x

[8] Verdín J, Bartnicki-García S, Riquelme M (2009) Functional stratification of the Spitzenkörper of *Neurospora crassa.* . Mol Microbiol 74: 1044–1053. 19843220 10.1111/j.1365-2958.2009.06917.x

[9] Pantazopoulou A, Peñalva MA (2009) Organization and dynamics of the *Aspergillus nidulans* Golgi during apical extension and mitosis. Mol Biol Cell 20: 4335–4347. 19692566 10.1091/mbc.E09-03-0254 PMC2762137

[10] Pantazopoulou A, Peñalva MA (2011) Characterization of *Aspergillus nidulans* RabC^Rab6^ . Traffic 12: 386–406. 21226815 10.1111/j.1600-0854.2011.01164.x

[11] Breakspear A, Langford KJ, Momany M, Assinder SJ (2007) CopA:GFP localizes to putative Golgi equivalents in *Aspergillus nidulans* . FEMS Microbiol Lett 277: 90–97. 17986089 10.1111/j.1574-6968.2007.00945.x

[12] Barlowe CK, Miller EA (2013) Secretory protein biogenesis and traffic in the early secretory pathway. Genetics 193: 383–410. 23396477 10.1534/genetics.112.142810 PMC3567731

[13] Kimura S, Maruyama J, Watanabe T, Ito Y, Arioka M, et al (2010) *In vivo* imaging of endoplasmic reticulum and distribution of mutant α-amylase in *Aspergillus oryzae* . Fungal Genet Biol 47: 1044–1054. 20884367 10.1016/j.fgb.2010.09.003

[14] English AR, Zurek N, Voeltz GK (2009) Peripheral ER structure and function. Curr Opin Cell Biol 21: 596–602. 19447593 10.1016/j.ceb.2009.04.004 PMC2753178

[15] Loewen CJ, Young BP, Tavassoli S, Levine TP (2007) Inheritance of cortical ER in yeast is required for normal septin organization. J Cell Biol 179: 467–483. 17984322 10.1083/jcb.200708205 PMC2064793

[16] Horio T, Oakley BR (2005) The role of microtubules in rapid hyphal tip growth of *Aspergillus nidulans.* . Mol Biol Cell 16: 918–926. 15548594 10.1091/mbc.E04-09-0798 PMC545922

[17] De Souza CP, Osmani AH, Hashmi SB, Osmani SA (2004) Partial nuclear pore complex disassembly during closed mitosis in *Aspergillus nidulans* . Current Biol 14: 1973–1984. 10.1016/j.cub.2004.10.050 15556859

[18] Osmani AH, Davies J, Liu HL, Nile A, Osmani SA (2006) Systematic deletion and mitotic localization of the nuclear pore complex proteins of *Aspergillus nidulans.* . Mol Biol Cell 17: 4946–4961. 16987955 10.1091/mbc.E06-07-0657 PMC1679664

[19] Wedlich-Soldner R, Schulz I, Straube A, Steinberg G (2002) Dynein supports motility of endoplasmic reticulum in the fungus *Ustilago maydis* . Mol Biol Cell 13: 965–977. 11907275 10.1091/mbc.01-10-0475 PMC99612

[20] Walter P, Lingappa VR (1986) Mechanism of protein translocation across the endoplasmic reticulum membrane. Ann Rev Cell Biol 2: 499–516. 3030381 10.1146/annurev.cb.02.110186.002435

[21] Rapoport TA (2007) Protein translocation across the eukaryotic endoplasmic reticulum and bacterial plasma membranes. Nature 450: 663–669. 18046402 10.1038/nature06384

[22] Panzner S, Dreier L, Hartmann E, Kostka S, Rapoport TA (1995) Posttranslational protein transport in yeast reconstituted with a purified complex of Sec proteins and Kar2p. Cell 81: 561–570. 7758110 10.1016/0092-8674(95)90077-2

[23] Deshaies RJ, Sanders SL, Feldheim DA, Schekman R (1991) Assembly of yeast Sec proteins involved in translocation into the endoplasmic reticulum into a membrane-bound multisubunit complex. Nature 349: 806–808. 2000150 10.1038/349806a0

[24] Gorlich D, Rapoport TA (1993) Protein translocation into proteoliposomes reconstituted from purified components of the endoplasmic reticulum membrane. Cell 75: 615–630. 8242738 10.1016/0092-8674(93)90483-7

[25] Jermy AJ, Willer M, Davis E, Wilkinson BM, Stirling CJ (2006) The Brl domain in Sec63p is required for assembly of functional endoplasmic reticulum translocons. J Biol Chem 281: 7899–7906. 16368690 10.1074/jbc.M511402200

[26] Cove DJ (1966) The induction and repression of nitrate reductase in the fungus *Aspergillus nidulans.* . Biochim Biophys Acta 113: 51–56. 5940632 10.1016/s0926-6593(66)80120-0

[27] Szewczyk E, Nayak T, Oakley CE, Edgerton H, Xiong Y, et al (2006) Fusion PCR and gene targeting in *Aspergillus nidulans.* . Nat Prot 1: 3111–3120. 10.1038/nprot.2006.405 17406574

[28] Tilburn J, Scazzocchio C, Taylor GG, Zabicky-Zissman JH, Lockington RA, et al (1983) Transformation by integration in *Aspergillus nidulans.* . Gene 26: 205–211. 6368319 10.1016/0378-1119(83)90191-9

[29] Nayak T, Szewczyk E, Oakley CE, Osmani A, Ukil L, et al (2005) A versatile and efficient gene targeting system for *Aspergillus nidulans.* . Genetics 172: 1557–1566. 16387870 10.1534/genetics.105.052563 PMC1456264

[30] Araujo-Bazán L, Peñalva MA, Espeso EA (2008) Preferential localization of the endocytic internalization machinery to hyphal tips underlies polarization of the actin cytoskeleton in *Aspergillus nidulans* . Mol Microbiol 67: 891–905. 18179595 10.1111/j.1365-2958.2007.06102.x

[31] Abenza JF, Pantazopoulou A, Rodríguez JM, Galindo A, Peñalva MA (2009) Long-distance movement of *Aspergillus nidulans* early endosomes on microtubule tracks. Traffic 10: 57–75. 19000168 10.1111/j.1600-0854.2008.00848.x

[32] Peñalva MA (2005) Tracing the endocytic pathway of *Aspergillus nidulans* with FM4–64. Fungal Genet Biol 42: 963–975. 16291501 10.1016/j.fgb.2005.09.004

[33] Zimmermann R, Eyrisch S, Ahmad M, Helms V (2011) Protein translocation across the ER membrane. Biochim Biophys Acta 1808: 912–924. 20599535 10.1016/j.bbamem.2010.06.015

[34] Ukil L, De Souza CCP, Liu HL, Osmani SA (2009) Nucleolar separation from chromosomes during *Aspergillus nidulans* mitosis can occur without spindle forces. Mol Biol Cell 20: 2132–2145. 19211837 10.1091/mbc.E08-10-1046 PMC2669022

[35] Upadhyay S, Shaw BD (2008) The role of actin, fimbrin and endocytosis in growth of hyphae in *Aspergillus nidulans* . Mol Microbiol 68: 690–705. 18331474 10.1111/j.1365-2958.2008.06178.x

[36] Travers KJ, Patil CK, Wodicka L, Lockhart DJ, Weissman JS, et al (2000) Functional and genomic analyses reveal an essential coordination between the unfolded protein response and ER-associated degradation. Cell 101: 249–258. 10847680 10.1016/s0092-8674(00)80835-1

[37] Guillemette T, van Peij NN, Goosen T, Lanthaler K, Robson GD, et al (2007) Genomic analysis of the secretion stress response in the enzyme-producing cell factory *Aspergillus niger.* . BMC Genomics 8: 158. 17561995 10.1186/1471-2164-8-158 PMC1894978

[38] Sims AH, Gent ME, Lanthaler K, Dunn-Coleman NS, Oliver SG, et al (2005) Transcriptome analysis of recombinant protein secretion by *Aspergillus nidulans* and the unfolded-protein response *in vivo.* . Appl Environ Microbiol 71: 2737–2747. 15870366 10.1128/AEM.71.5.2737-2747.2005 PMC1087583

[39] Saloheimo M, Valkonen M, Penttila M (2003) Activation mechanisms of the HAC1-mediated unfolded protein response in filamentous fungi. Mol Microbiol 47: 1149–1161. 12581366 10.1046/j.1365-2958.2003.03363.x

[40] Valdez-Taubas J, Pelham HR (2003) Slow diffusion of proteins in the yeast plasma membrane allows polarity to be maintained by endocytic cycling. Current Biol 13: 1636–1640. 10.1016/j.cub.2003.09.001 13678596

[41] Lee SC, Shaw BD (2008) Localization and function of ADP ribosylation factor A in *Aspergillus nidulans.* . FEMS Microbiol Lett 283: 216–222. 18430001 10.1111/j.1574-6968.2008.01174.x

[42] Harris SD (2006) Cell polarity in filamentous fungi: shaping the mold. Int Rev Cytol 251: 41–77. 16939777 10.1016/S0074-7696(06)51002-2

[43] Gladfelter AS (2010) Guides to the final frontier of the cytoskeleton: septins in filamentous fungi. Curr Opin Microbiol 13: 720–726. 20934902 10.1016/j.mib.2010.09.012

[44] Hernández-Rodríguez Y, Hastings S, Momany M (2012) The septin AspB in *Aspergillus nidulans* forms bars and filaments and plays roles in growth emergence and conidiation. Eukaryot Cell 11: 311–323. 22247265 10.1128/EC.05164-11 PMC3294440

[45] Lindsey R, Cowden S, Hernández-Rodríguez Y, Momany M (2010) Septins AspA and AspC are important for normal development and limit the emergence of new growth foci in the multicellular fungus *Aspergillus nidulans.* . Eukaryot Cell 9: 155–163. 19949047 10.1128/EC.00269-09 PMC2805303

[46] Puhka M, Vihinen H, Joensuu M, Jokitalo E (2007) Endoplasmic reticulum remains continuous and undergoes sheet-to-tubule transformation during cell division in mammalian cells. J Cell Biol 179: 895–909. 18056408 10.1083/jcb.200705112 PMC2099207

[47] Prescott AR, Farmaki T, Thomson C, James J, Paccaud JP, et al (2001) Evidence for prebudding arrest of ER export in animal cell mitosis and its role in generating Golgi partitioning intermediates. Traffic 2: 321–335. 11350628 10.1034/j.1600-0854.2001.002005321.x

[48] Straube A, Weber I, Steinberg G (2005) A novel mechanism of nuclear envelope break-down in a fungus: nuclear migration strips off the envelope. EMBO J 24: 1674–1685. 15861140 10.1038/sj.emboj.7600644 PMC1142577

[49] Riquelme M, Fischer R, Bartnicki-Garcia S (2003) Apical growth and mitosis are independent processes in *Aspergillus nidulans.* . Protoplasma 222: 211–215. 14714210 10.1007/s00709-003-0020-8

